# Editorial: Anti-cancer bioactive molecules from microbial sources

**DOI:** 10.3389/fphar.2023.1190354

**Published:** 2023-04-13

**Authors:** Bishwambhar Mishra, Saravanan Muthupandian, Hamed Barabadi, Kumananda Tayung, Yugal Kishore Mohanta

**Affiliations:** ^1^ Department of Biotechnology, Chaitanya Bharathi Institute of Technology, Hyderabad, Telengana, India; ^2^ AMR and Nanomedicine Lab, Department of Pharmacology, Saveetha Dental College, Saveetha Institute of Medical and Technical Sciences (SIMATS), Chennai, India; ^3^ Department of Pharmaceutical Biotechnology, School of Pharmacy, Shahid Beheshti University of Medical Sciences, Tehran, Iran; ^4^ Department of Botany, Gauhati University, Guwahati, Assam, India; ^5^ Nanobiotechnology and Translational Knowledge Laboratory, Department of Applied Biology, School of Biological Sciences, University of Science and Technology Meghalaya (USTM), Baridua, Meghalaya, India

**Keywords:** bioactive molecules, immunotherapy, antiproliferative effects, toxicity evaluation, targeted delivery

Cancer incidence and mortality have historically been a source of communal concern. Cancer is one of the world’s leading causes of death, with a projected annual mortality rate of 16.4 million by 2040. In general, its mortality rate has decreased over the years; however, there is still an increased mortality rate in poorer countries where healthcare professionals pay special attention. About 200 types of cancer affect humans, and for the majority of these, just a handful of approved treatments exist (Law et al., 2020).

To prevent cancer-related mortality, it is essential to develop novel, strong anticancer drugs. The discovery of anti-cancer drugs has witnessed significant technological advancement in recent years. Traditional cell-based screening for antiproliferative effects has been replaced with a particular technique to scan for compounds that can target major cancer proteins or pathways (Izadi et al., 2020).

In recent decades, the hunt for bioactive compounds from microbes has garnered increasing interest. Numerous anticancer medicines generated from actinobacteria groups of microorganisms (e.g., *Streptomyces parvulus*, *Streptomyces antibioticus*, *Streptomyces cheonanensis*, *Streptomyces anandii*, etc.) have been studied in clinical trials. Notable examples of anti-cancer bioactive chemicals include actinomycin D, bleomycin, anthracyclines, epirubicin, and doxorubicin (Silva et al., 2020). One of the most exciting areas of study for treating the cancer cells is immunotherapy, which can involve the application of particular microbes (e.g., *Alistipes shaii*, *Bacteroides fragilis*, *Bifidobacterium*, *Faecalibacterium,* etc.). The immune system is stimulated through T cell responses to microbial antigens that increase antitumor immunity with tumor-specific antigens, or through small metabolites that mediate systemic effects on the host by binding to PRRs (Pattern Recognition Receptors) (Inamura, 2020). In response to bacterial stimulation (toxin production, binding substances that can be delivered to a specific location (vectors), and anaerobic lifestyle), there is an intense activation (increasing the number and recruitment of neutrophils, monocytes/macrophages, natural killer cells, etc.) of the immune system in the host cell (Łukasiewicz and Fol, 2018).

This Research Topic gives a deeper understanding of the development, characteristics, and mechanistic studies of microorganism-derived anti-cancer compounds. Under the subject theme, three experimental research publications and two review articles have been published.

Li et al. did an interesting study on how calf thymus polypeptide (CTP) stops colorectal cancer (CRC) in B6/JGpt-*Apc* ^em1Cin (MinC)^/Gpt mice by controlling their intestinal microbiota and immune response. The immune system is altered by CTP via the intestinal micro biota, which is linked to the drug’s anti-CRC effect. This establishes a connection between CTP and the use of therapeutic drugs or drug combinations in a clinical setting for the management of CRC. CTP makes CRC less likely to happen because it helps the immune system work well through the Interleukin-2 (IL-2)-associated signaling pathway. Even though this current topic confirmed that CTP associated with T cells has an effect on CRC that slows it down, more research is needed to find out how it changes and be sure it works in the clinical trials.

Another noteworthy study in this Research Topic by Zhang et al. claims that synergistic effects of *Pseudomonas aeruginosa* and ambient particulate matter with a diameter of 2.5 m (PM 2.5) disrupt alveolar macrophage function and cause inflammation. Moreover, PM 2.5 and *P. aeruginosa* were used to excite alveolar macrophages in an enclosed broiler house, whereas PM 2.5 in combination with *P. aeruginosa* was found to reduce phagocytosis, block autophagy, promote apoptosis, and damage the cytoskeleton in alveolar macrophages. Furthermore, alveolar macrophages significantly increased production of mTOR pathway-related proteins in response to the synergistic stimulation of PM 2.5 and *P. aeruginosa*.

A study by Fayek et al. examined the anticancer properties of several metabolites that were initially identified from *Aspergillus neoniveus*, such as terpenoids, alkaloids, γ-butyrolactone, quinoids, nervonic methyl ester, acetylaszonalenin, and butyrolactone II, towards prostate cancer. These isolated metabolites significantly inhibited human prostate cancer cell invasion, metastasis, and proliferation in animal models at micro molar concentrations. Computational target prediction methods also uncovered cannabinoid G-protein coupled receptor type 1 (CB1) as a putative biological target mediating, at least in part, the anticancer actions of acetylaszonalenin.

Omole et al. investigate the field of oncolytic viruses, which have become a viable immunotherapeutic choice for the treatment of many tumors. In this review, they have looked at the primary traditional therapeutic methods for the treatment of cancer and each one’s specific shortcomings, as well as how severe the burden of cancer is on a global scale, especially in sub-Saharan Africa. Several pre-clinical and clinical trials have used the alleged mechanisms of action of oncolytic viruses and other viruses that have found application in the fight against various types of oncolytic viruses to treat cancer. The use of oncolytic viro-immunotherapy for the treatment of malignancies has raised concerns about toxicity and safety. This study also looked at the anticipated future directions for researchers and the general public who desire updated information.

Li et al. look at the latest research on cell penetrating peptides (CPPs), peptide-based vaccinations, and anticancer peptides for treating and preventing breast cancer. Moreover, different types of peptides that work as targeted nanovectors, cancer vaccines, and anti-cancer drugs to treat breast cancer were looked at in detail. Anticancer treatment using microorganisms is usually ignored or marginalized. A very small group of scientists explores and creates cancer treatment plans that make use of microorganisms, either as vaccinations that amplify the immune system’s capacity to combat disease or as delivery systems for anticancer drugs. Despite important developments in immunotherapy, these findings are occasionally disregarded. Animals were employed in experimental studies to show that the most successful strain removed cancer, but the animals perished from infection with diseases. The safety of the patients must be guaranteed, notably by using only microorganisms that have undergone the necessary attenuation. The desired effect can only be ensured by a precise balance between a microorganism’s attenuation and its capacity to excite the immune system.

The authors and reviewers of these manuscripts are to be commended for their hard work and dedication. Moreover, the reviewers are highly appreciated for their helpful remarks that enhanced the quality of the manuscripts.

Our objective is that this compilation of articles will serve as a resource for researchers from a wide range of disciplines who are interested in evaluating anti-cancer bioactive molecules from microbial sources in their quest to develop novel pharmacological approaches.
